# Effectiveness of different types of ultrasonography screening for developmental dysplasia of the hip: A meta-analysis

**DOI:** 10.1097/MD.0000000000023562

**Published:** 2020-12-11

**Authors:** Hae Woon Jung, Woo Young Jang

**Affiliations:** a Department of Pediatrics, Kyung Hee University Medical Center; b Department of Orthopedic Surgery, College of Medicine, Korea University, Seoul, Korea.

**Keywords:** developmental dysplasia of the hip, selective hip ultrasonography screening, universal hip ultrasonography screening

## Abstract

**Background:**

Infant hip screening for early detection of developmental dysplasia of the hip (DDH) is essential as early detection can enable less invasive treatments and achieve better long-term results. A previous meta-analysis assessed about 10,000 infants per group, which is insufficient for evaluating the effect of different infant hip screening strategies on early detection and treatment of DDH. Therefore, we conducted a systematic review and meta-analysis using both randomized controlled trials and cohort studies to determine the effects of universal hip ultrasonography screening (UHUS) and selective hip ultrasonography screening (SHUS) on the incidence of late-diagnosed DDH.

**Methods:**

A literature search of PubMed, EMBASE, and Cochrane databases was performed. The summary odds ratio (OR) with 95% confidence interval (CI) was calculated using fixed-effects models.

**Results:**

Meta-analysis of five studies that met the eligibility criteria revealed a significant difference in late-diagnosed DDH (OR 0.44, 95% CI 0.23–0.83) between infants screened using UHUS (n = 29,070) and those screened using SHUS (n = 30,442) in a fixed-effects model without heterogeneity among studies. In the subgroup analysis, meta-analysis of the randomized controlled trials showed no significant difference in late-diagnosed DDH (OR 0.52, 95% CI 0.20–1.39) between infants screened using UHUS (n = 11,453) and those screened using SHUS (n = 12,077) in a fixed-effects model with low heterogeneity among studies (I^2^ = 0.9%). However, meta-analysis of the cohort studies showed a significant difference in late-diagnosed DDH (OR 0.38, 95% CI 0.17–0.89) between infants screened using UHUS (n = 17,617) and those screened using SHUS (n = 18,345) in a fixed-effects model with low heterogeneity among studies. Sensitivity analysis revealed that the impact of each study on the summary results was not significant. There was no publication bias in our meta-analysis.

**Conclusions:**

Our meta-analysis suggests that a statistically significant decrease in the incidence of late-diagnosed DDH is possible when UHUS is adopted compared with SHUS. Our study provides information about the effects of different infant hip screening strategies on the incidence of late-diagnosed DDH, which can help decide upon which strategy to apply.

## 1 Background

Early detection of developmental dysplasia of the hip (DDH) is essential as it allows for the implementation of less invasive treatment methods and the achievement of good long-term results.^[1,2]^ Therefore, some countries have adopted a newborn hip screening program for the detection of DDH. Ultrasonography is accepted as a reliable tool for early detection of DDH because it is noninvasive and can identify problems in the cartilaginous hip much earlier than radiography.^[3]^ Ultrasonography screening programs for DDH may be universal, with all newborns being screened, or selective, with only infants with risk factors for DDH or with abnormalities detected upon physical examination of the hip being screened. There have been reports of decreased surgical treatment for DDH after implementation of universal ultrasonography screening for DDH in Germany and Austria.^[4]^ However, the effectiveness of universal hip ultrasonography screening (UHUS) over selective hip ultrasonography screening (SHUS) has not yet been established.

Given that the incidence of DDH is approximately 1.4 to 20 per 1000 newborns, a large number of patients are needed to successfully evaluate the effect of screening strategies on early detection. Krismer et al^[5]^ advocated that, based on infant hip screening strategies, each group would have to consist of 20,000 infants to ensure a significance level of 0.05. However, in a previous meta-analysis,^[4]^ the sample size was about 10,000 infants per group, which may have been insufficient for analysis. We hypothesized that a large study size could possibly bypass the limitations of the previous meta-analysis, which did not have sufficient power to determine differences. Therefore, in the present study, we conducted a systematic review and meta-analysis using both randomized controlled trials (RCTs) and cohort studies to determine the effects of different infant hip screening strategies (UHUS and SHUS) on the incidence of late diagnosis of DDH.

## 2 Methods

### 2.1 Search strategy

Institutional review board approval and patient consent were not required since this study was a meta-analysis. A literature search was conducted using PubMed, MEDLINE (January 1950–January 2020), EMBASE (January 1966– January 2020), and Cochrane Library databases (January 1960–January 2020). The reference lists of the original studies were manually searched. We searched the databases using the following text words and/or medical subject heading terms: “sonography” or “dysplasia” or “ultrasound” and “hip” and “screening.” The articles were restricted to English because of a lack of accessibility and comprehension in other languages. The titles and abstracts of eligible citations were screened. Selected articles were evaluated independently, and disagreements were resolved consensually. Bibliographies of systematic reviews and all the articles of the authors who reported on hip ultrasonography screening were manually reviewed to retrieve articles not captured by the initial database search.

### 2.2 Study selection

Based on the title and abstract, 2 reviewers independently selected relevant studies for full review. The full-text copy of the article was reviewed if the abstract did not provide enough data on which to make a decision. Studies that met the inclusion criteria for meta-analysis had the following characteristics:

(1) retrospective or prospective comparison of late-diagnosed DDH between UHUS and SHUS strategies;
(2) fully reported number of subjects in each group (UHUS and SHUS groups);
(3) use of adequate statistical methods to compare these parameters between groups. Studies were excluded if
(4) they had missing or inadequate outcome data, or
(5) they were case series, non-clinical studies (i.e., basic science, cadaveric, biomechanical), expert opinions, reviews, commentaries, economic-decision analyses, editorials, and not written in English.

### 2.3 Quality assessment

Two authors (Jang WY and Lee SH) independently performed data extraction and quality assessment using a data extraction form. Quality assessment was also performed independently by reviewers using the Cochrane Risk of Bias (RoB)^[6]^ for RCTs and risk of bias assessment tool for nonrandomized studies (RoBANS 2.0)^[7]^ for nonrandomized comparative studies. RoB has 7 domains and RoBANS 2.0 has 8 domains to assess the methodological quality of studies. Each criterion was evaluated as “low risk of bias,” “high risk of bias,” or “unclear.” If the study did not mention a certain criterion, we evaluated it as “unclear.” At each instance of disagreement, the case was discussed by all authors.

### 2.4 Extraction of data

The following data were extracted from the selected studies: institution and country of the study; year of publication; number, sex, and age of the patients; and definition of late-diagnosed DDH. All disagreements were resolved consensually.

### 2.5 Outcome measures

Routine screening for DDH aims to eliminate late diagnosis of infants with established subluxation and dislocation. Such late diagnoses frequently result in the need for major operations, which often result in unsatisfactory long-term outcomes, development of early degenerative changes in the hip, and functional morbidity. Therefore, incidence of late-diagnosed DDH was considered the outcome of ultrasonography screening. We considered the odds ratio (OR) of each study as the effect size.

### 2.6 Statistical analysis

We used Higgins I^2^ statistics to determine the percentage of total variation across studies owing to heterogeneity. The value of I^2^ ranges from 0% (no observed heterogeneity) to 100% (maximal heterogeneity); I^2^ > 50% may be considered to represent substantial heterogeneity. A pooled OR was analyzed using an inverse variance weighting method, and the fixed-effects model was selected on the basis of heterogeneity. A forest plot has been used to display the meta-analysis data. The point estimate for risk ratio is represented by a square, and the confidence interval (CI) for each study is represented by a horizontal line. The size of the square corresponds to the weight of the study in the meta-analysis, with larger shapes assigned to studies with larger sample sizes, better quality data, or both. Sensitivity analysis was employed to determine the influence of each individual study on the summary results by repeating random-effects meta-analysis, omitting one study at a time. For identifying publication bias, Begg funnel plot was used. All statistical analyses used in this study were performed using R v3.1.2 (metafor packages). *P* < .05 was considered statistically significant.

## 3 Results

### 3.1 Included studies

**Figure 1:**
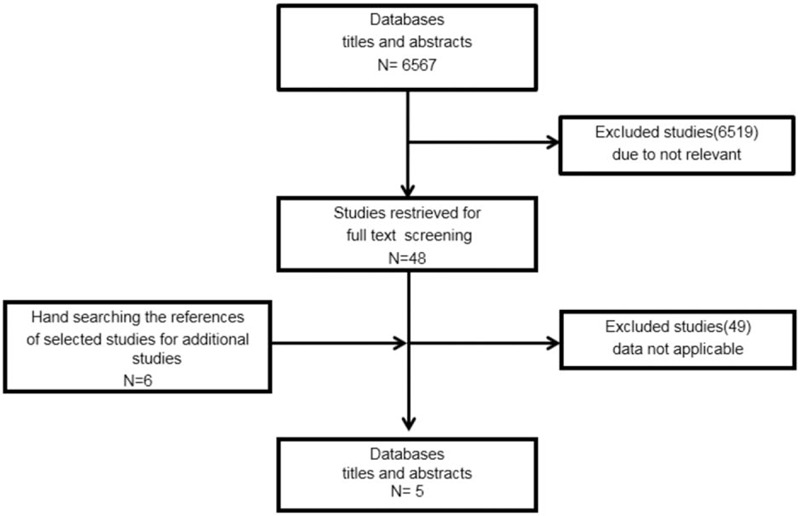
Flow diagram of the study selection process.

Literature searches of the three electronic databases using the search terms mentioned earlier identified 6567 publications. All studies retrieved from the databases were independently evaluated. After reviewing the abstracts and/or titles, 48 potentially relevant publications were identified for further full-text examination. By searching the reference lists of 48 relevant publications, 6 additional reports were included to obtain a total of 54 full-text examinations. Of these, 49 did not have adequate data for meta-analysis and were excluded. Manual searching led to the inclusion of 1 study for meta-analysis. Finally, a total of five studies were included in the meta-analysis (Fig. 1).

### 3.2 Study characteristics and quality assessment

The selected studies were published between 1989 and 2004, with reports on a total of 59,492 infants. The eligible studies consisted of 2 RCTs and 3 cohort studies. The sample size ranged from 2121 to 18,669 patients. Only 1 of the 5 studies reported a significant difference in the incidence of late-diagnosed DDH between UHUS and SHUS. Four studies were conducted at a single institution, and 1 study reported local and regional data. Data from the local/regional study by Clegg et al^[8,9]^ was collected from 2 separately published reports. Outcomes of hip screening strategies that changed over time in the Coventry area of United Kingdom (UK) were reported. This area adopted SHUS as the hip screening strategy from January 1986 to June 1989 and UHUS thereafter (from July 1989). This meta-analysis included the incidence of late DDH in 1986 for SHUS and late DDH incidence between June 1989 and December 1996 for UHUS (Table 1).

**Table 1:** Characteristics of the eligible studies.

Study, yr	Trial type	Country	Participants	Late-diagnosed DDH	Screening
Holen, 2002	Randomized controlled trial	Norway	16,629 newborns at a single center born 1988–92	> 1 mo	Group 1 (n = 7840): general clinical screening plus ultrasound screening group 2 (n = 7689): general clinical screening plus selective use ultrasonography
Rosendahl, 1994	Randomized controlled trial	Norway	11,925 newborns at a single hospital born 1988–90	> 1 mo	Group 1 (n = 3613): universal ultrasound. group 2 (n = 4388): selective ultrasound (if clinical dislocation, dislocatable or instability, breech, close family history of DDH)
Stover, 1992	Cohort study	Germany	2121 newborns at a single hospital 1988–92	> 9 wk	Group 1 (n = 726): universal ultrasound. group 2 (n = 1395): selective ultrasound (if clinical reason or family history of DDH)
Clegg 1989,1999	Cohort study	United Kingdom	18,669 newborns in Coventry born 1976–96	> 6 wk	Group 1 (n = 4619): general clinical screening plus selective use of ultrasonography 1986 group 2 (n = 14050): general clinical screening 1989-96
Wirth, 2004	Cohort study	Germany	12,331 newborns at three hospitals born 1985–98	> 6 mo	Group 1 (n = 12331): general clinical screening plus selective use of ultrasonography group 2 (n = 2841): general clinical screening 1989-96

**Figure 2:**
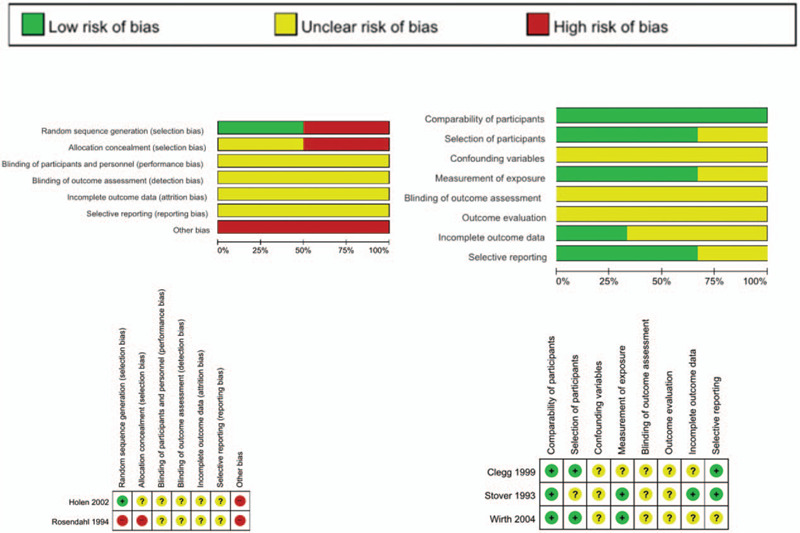
Quality assessment of the included studies. Quality assessment was performed using the Cochrane risk of bias (RoB) for randomized controlled trials and the risk of bias assessment tool for nonrandomized studies (RoBANS 2.0). Green represents low risk of bias; yellow represents unclear risk; and red represents high risk of bias.

The definition of late-diagnosed DDH varied among the studies. Holen et al^[10]^ and Rosendahl et al^[11]^ defined late diagnosis of DDH as when an infant presented with DDH 1 month after birth, while Clegg et al^[8,9]^ defined DDH as cases presenting with DDH after 6 weeks; Wirth et al^[12]^ defined it as after 9 weeks, and Stöver et al^[13]^ defined late diagnosis as those cases presenting with DDH after 6 months. The risk of bias in the RCTs was poor; however, the cohort studies were of high quality (Fig. 2).

### 3.3 Data synthesis and review

**Figure 3:**
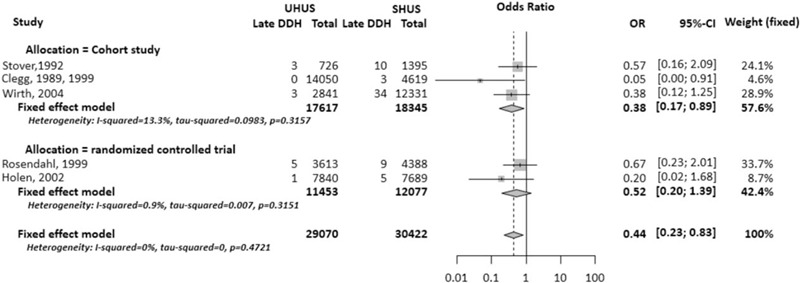
Forest plot and meta-analysis.

Meta-analysis of the 5 studies showed a significant difference in late-diagnosed DDH (OR 0.44, 95% CI 0.23–0.83) between infants screened using UHUS (n = 29,070) and those screened using SHUS (n = 30.442) in a fixed-effects model without heterogeneity among studies (Fig. 3). In the subgroup analysis, meta-analysis of the RCTs showed no significant difference in late-diagnosed DDH (OR 0.52, 95% CI 0.20–1.39) between infants screened using UHUS (n = 11,453) and those screened using SHUS (n = 12,077) in a fixed-effects model with low heterogeneity among studies (I^2^ = 0.9%). However, meta-analysis of the cohort studies showed a significant difference in late-diagnosed DDH (OR 0.38, 95% CI 0.17–0.89) between infants screened using UHUS (n = 17,617) and those screened using SHUS (n = 18,345) in a fixed-effects model with low heterogeneity among studies (I^2^ = 13.3%).

### 3.4 Sensitivity analysis and publication bias

**Figure 4:**
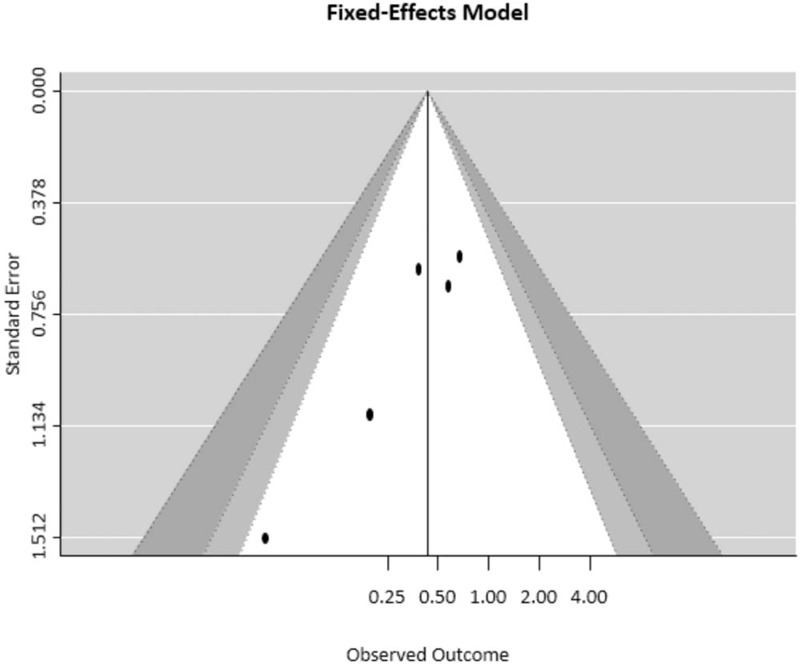
Sensitivity analysis showed that the impact of each study on the summary results was not significant.

**Figure 5:**
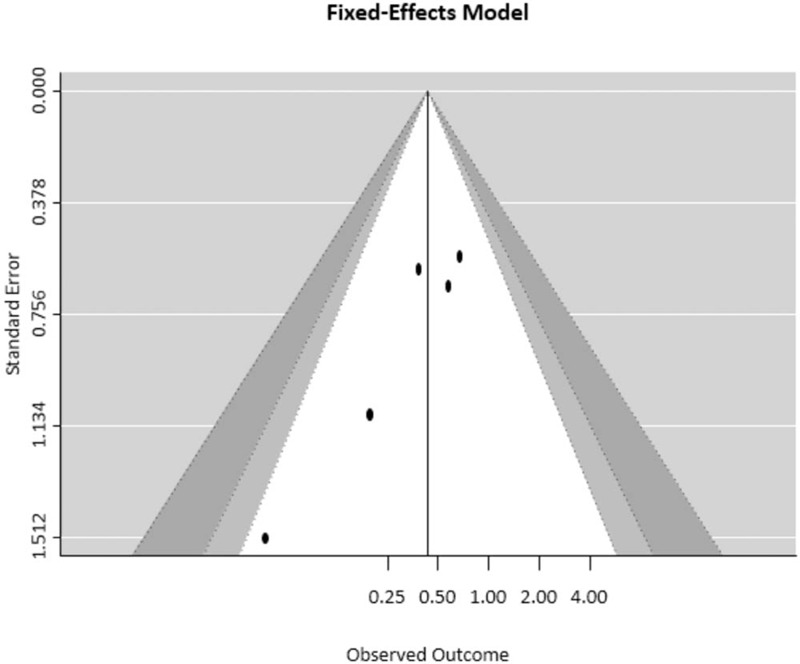
Funnel plot of the studies used in the meta-analysis.

Sensitivity analysis revealed that the impact of each study on the summary results was not significant (Fig. 4). Funnel plots were used to estimate the publication bias of the included literature. The shapes of the funnel plots revealed that the included studies had no apparent asymmetry (Fig. 5).

## 4 Discussion

The aim of infant hip screening strategies is the early identification of all cases of hip instability and dysplasia. Although clinical screening for DDH is considered effective in the presence of structural or functional abnormalities, it is difficult to detect mild dysplastic DDH. For these reasons, many authors advocate the need for ultrasound screening for DDH.^[3]^ SHUS is a strategy in which ultrasound imaging is selectively performed in infants who show positive findings during clinical screening or have significant risk factors such as breech position or family history of DDH. In contrast, UHUS is universally performed in all newborns so as to not to miss a diagnosis of DDH.^[4]^ UHUS is associated with a reduction in the number and severity of surgical interventions related to DDH.^[14]^ The rate of late-diagnosed DDH is commonly used as an outcome in the evaluation of screening strategies. Undetected or late diagnosis of DDH leads to unfavorable prognosis for the patient and family.^[15,16]^ A dysplastic hip that is not diagnosed on time, left untreated, or treated improperly may result in limping, leg length discrepancy, pain, frequent operations, femoral head necrosis, osteoarthritis, disability, and even total hip replacement at a young age.^[17]^ The efficacy of ultrasonography in reducing the frequency of missed diagnoses of DDH and increasing early detection is well known.^[18,19]^ However, there is some controversy over whether UHUS or SHUS is the better screening method for this purpose.^[4,9,14]^ Analysis of both RCTs and non-RCTs in our meta-analysis supports the hypothesis that UHUS can significantly reduce the incidence of late-diagnosed DDH compared with SHUS.

The implications of medical research need to be carefully reviewed as results on the same intervention could be drastically different, or even contradictory, depending on the study type. Furthermore, although an intervention may, in fact, yield better outcomes, the effect may not be statistically significant owing to small sample sizes. In such cases, it is difficult to apply and generalize the results directly to patients. Meta-analysis is a method of scientific and statistical integration of results from a series of individual studies which can provide important insights for application of medical research to patient care. Since systematic reviews and meta-analyses are affected by the quality of the included articles, assessment of the quality of primary studies is important to minimize the potential for biased estimates of intervention effects.^[20]^ Quality assessment is especially important when including both RCTs and nonrandomized studies in meta-analysis.^[21]^ In this meta-analysis, the included RCTs showed high risks of bias, whereas the included cohort studies showed high quality. In such a situation, some authors^[21–25]^ advocate the inclusion of both RCTs and non-RCTs in the analysis, as non-RCT studies offer advantages of longer follow-up time, larger sample size, real-world data, and more generalizable findings.^[23]^ RCTs, in comparison, may have limitations of short follow-up time, small sample size, a highly selected population, high cost, and ethical constraints for studying certain treatments or populations.^[22]^ Analysis of both RCTs and observational studies in this meta-analysis increased the sample size and resulted in a reliably summarized effect size, the results of which indicated that UHUS showed significantly less risk of late diagnosis of DDH.

Several studies have reported rates of late-diagnosed DDH.^[11,13–15]^ However, interpretation of late-diagnosed DDH is often difficult because of inconsistencies in the definition of age at diagnosis. The cut-off age for late diagnosis of DDH in literature can vary from 6 weeks to 20 months. Moreover, the rate of late-diagnosed DDH, according to previous reports, varies from 0.07 to 2.0 per 1000 births.^[26]^ In this meta-analysis, the definition of late-diagnosed DDH also varied slightly among the studies. However, because the primary outcome of this study was not the incidence of late-diagnosed DDH but the OR, the bias as per the variations in the definition of late-diagnosed DDH was not expected to be high. Furthermore, the values of OR in the 2 cohort studies by Stöver et al and Wirth et al were 0.57 and 0.37, respectively, which were between the ORs of the 2 RCTs by Holen et al and Rosendahl et al. In comparison, the OR of the study by Clegg et al^[8,9]^ was smaller at 0.04. This may be owing to the high efficacy of well-established UHUS in the Coventry area of UK. Their results had only a small influence on summarized OR (weight: 4.5%), and further sensitivity analysis, even after exclusion of the study by Clegg et al, continued to show a significantly decreased summarized OR for late-diagnosed DDH with UHUS.

Several authors have claimed that clinical screening by well-trained and experienced doctors when accompanied by SHUS will have effects similar to those of UHUS on reducing the rate of late-diagnosed DDH.^[15,27]^ However, there is no consensus on the definition of “well-trained” and “experienced.” Due consideration must be given to the fact that even though the authors of the 2 RCTs^[10,11]^ in this study were experts in DDH detection, the incidence of late-diagnosed DDH with UHUS was lower than that with SHUS, although not significantly. Clinical examination for DDH is often subjective, and even fully dislocated joints may sometimes be missed and not detected. Additionally, more than 50% of DDH cases do not have any “typical” risk factors.^[28]^ Furthermore, according to a previous report,^[29]^ late-diagnosed DDH is more prevalent in children without risk factors, implying that those with risk factors are assessed more closely at birth and identified more promptly. Thaler et al^[30]^ reported a significant reduction in the rate of surgery for DDH later in life after the introduction of universal ultrasound screening in Austria. Therefore, infant hip screening with ultrasonography, which is objective and reproducible with measurable results and does not involve radiation exposure, may be required for the early detection of DDH.

Several requirements must be met to maximize the benefits of UHUS. First, the timing of ultrasound screening should be after 6 weeks of age to prevent over-diagnosis and unnecessary treatment, as hip dysplasia in infants less than 4 weeks often reverts normally without treatment.^[31]^ Second, ultrasonography should be performed by well-trained doctors^[32]^ who are accurate and use objective methods such as the Graf^[33]^ or Harcke^[34]^ methods. Harcke et al^[35]^ suggested that qualifications for adequate hip ultrasonography should include previous experience with hip ultrasonography in at least 100 infants less than 6 months of age. Third, the cost of UHUS should be acceptable in the context of the national healthcare system,^[9,36]^ as the cost-effectiveness of UHUS of increased sensitivity is still a matter of debate.

Our meta-analysis has limitations that affect the interpretation of the true results. Several studies in this meta-analysis were retrospective, and are more susceptible to selection biases than RCTs. There is a need for additional high quality RCTs; when a sufficient number of primary studies becomes available for analysis, another systematic review and meta-analysis should be conducted and compared with this one. Second, in the present study, all the included studies were conducted in Europe. The regional concentration of a study population commonly influences generalizability and makes it difficult to interpret the results in the context of other countries.

## 5 Conclusion

This meta-analysis suggests that a statistically significant decrease in the incidence of late-diagnosed DDH is possible when UHUS is adopted compared with SHUS. However, the strategy of infant hip screening that is appropriate should be considered individually, by each country, in the context of socioeconomic factors and healthcare policies, including insurance. Our study may provide useful information on the effectiveness of infant hip screening in preventing late-diagnosed DDH.

## Author contributions

**Conceptualization:** Hae Woon Jung, Woo Young Jang.

**Data curation:** Hae Woon Jung.

**Funding acquisition:** Woo Young Jang.

**Methodology:** Woo Young Jang.

**Resources:** Woo Young Jang.

**Validation:** Hae Woon Jung.

**Visualization:** Hae Woon Jung.

**Writing – original draft:** Hae Woon Jung, Woo Young Jang.

**Writing – review & editing:** Hae Woon Jung, Woo Young Jang.
